# The Story of the Insane from Year to Year

**Published:** 1905-04-08

**Authors:** 


### THE STORY OF THE INSANE FROM YEAR TO YEAR.

Seventeenth Annual Series.

### THE LANCASHIRE COUNTY ASYLUM AT PRESTWICH.

On the first of January this asylum contained the enormous number of 2,693 patients, and at the end of December the number was 2,682. There were 589 admissions, 72 readmissions, 268 recoveries, 159 discharged relieved, and 242 deaths. The average number resident during the year was 2,675, and the total number under treatment during the year was 3,338. Calculated on the admissions the recoveries reached 40·54 per cent., and calculated on the average daily number resident the deaths were 7·22 per cent. The former percentage was above the average, and the latter below it. Of the year's deaths 82 were caused by cerebral and spinal diseases, 41 by pneumonia, 59 by consumption, 11 by other lung diseases, and one by colitis. Of the year's admissions 139 owed their insanity to such moral causes as domestic trouble, mental anxiety, and adverse circumstances; while among the physical causes drink accounts for 152, hereditary tendency for 133, and 133 are of unknown origin. The pressure on the accommodation has been very great, and 80 chronic patients have been sent to the Rochdale workhouse, an arrangement which has worked satisfactorily so far, but how long will it continue? Dr. Perceval says that the new asylum at Winwick has afforded relief to the pressure, but only to a limited extent. He thinks it very doubtful whether chronic asylums placed at a distance from their base are advisable, and we agree that much may be said against them, and we further agree that the accommodation for chronic cases should always be in the immediate neighbourhood of the acute asylum. But we are in total disagreement with Dr. Perceval when he speaks in favour of such monster asylums as those for Lancashire, and we greatly doubt whether these huge institutions can be managed so economically as smaller ones. To us there are objections which weigh heavier than merely economical ones, and we are strongly of opinion that no asylum should ever be built for more than 1,000 patients. That number is ample for all purposes of classification, and only in most exceptional circumstances should enlargement be permitted as high as 1,200 beds. We notice in this report that as far as possible only acute cases are now being admitted to the Prestwich asylum, and if Dr. Perceval were to devote an average of 15 seconds a day to each patient it would take him over 11 hours to see his cases. The clerk of the asylum retired during the year on a pension of £226, and Attendant Wilkinson received a pension of £52, and Carpenter Booth one of £26. The weekly charge per head is 9s. 11d. for in-county cases, 14s. for out-county paupers, and from 15s. to 21s. for private patients.

